# Anatomical and functional studies of vestibular neuroepithelia from patients with Ménière's disease

**DOI:** 10.1242/dmm.052224

**Published:** 2025-04-15

**Authors:** Hannah R. Drury, Melissa A. Tadros, Robert J. Callister, Alan M. Brichta, Robert Eisenberg, Rebecca Lim

**Affiliations:** ^1^ School of Biomedical Sciences and Pharmacy, The University of Newcastle, Callaghan, NSW 2308, Australia; ^2^ Brain Neuromodulation Program, Hunter Medical Research Institute, New Lambton Heights, NSW 2305, Australia; ^3^ School of Medicine and Public Health, The University of Newcastle, Callaghan, NSW 2308, Australia

**Keywords:** Ménière's disease, Vestibular, Hair cells, Immunofluorescent labelling, Electrophysiological recording

## Abstract

Surgical removal of vestibular end organs is a final treatment option for people with intractable Ménière's disease (MD). Here, we used surgically excised vestibular neuroepithelium from patients with MD for (1) anatomical investigation of hair cell and nerve fibre markers using immunohistochemistry, and (2) functional studies using electrophysiological recordings of voltage-activated currents. Our data show considerable reduction in and disorganisation of vestibular hair cells in the cristae ampullares. Nerve fibres maintain contact with remaining sensory receptors but appear thin in regions in which hair cells are absent. Electrophysiological recordings of voltage-activated potassium currents from surviving hair cells demonstrated normal activity in both type I and type II vestibular hair cells. Current-voltage plots from type I vestibular hair cells are consistent with the presence of a surrounding calyx afferent terminal. These data indicate that the surviving hair cells that were sampled in patients with MD remain functional and capable of transmitting sensory information to the central nervous system. Determining functionality of vestibular receptors and nerves is critical for vestibular implant research to restore balance in people with MD.

## Teaser

**Summary:** Electrophysiological recordings of surviving vestibular hair cells from Ménière's disease patients show normal voltage-activated currents, while immunofluorescent labelling of these cells shows disorganised neuroepithelia.

## INTRODUCTION

Ménière's disease (MD) is an intractable condition of the inner ear, affecting both balance and hearing. MD most often affects one ear, although in 20% of MD cases it can be bilateral (Huang and Young, 2015). Diagnosis of MD typically occurs some time after the onset of symptoms, and MD is classified into two categories – definite and probable MD – by the International Classification of Vestibular Disorders. Definite MD is diagnosed by an observation of episodic vertigo syndrome (20 min to 12 h), with low to medium frequency sensorineural hearing loss and fluctuating aural symptoms (hearing, tinnitus and/or fullness). Probable MD is defined as episodic vestibular symptoms (vertigo or dizziness), accompanied by fluctuating aural symptoms occurring in a period from 20 min to 24 h (Lopez-Escamez et al., 2015). MD onset typically occurs between 40 and 60 years of age, with a slightly increased incidence in women (Kim and Cheon, 2020).

MD can be bilateral or unilateral, with each of these having five distinct clinical subtypes (Frejo et al., 2016, 2017). Cluster analysis has identified that these clinical subtypes differ based on the presence or absence of associated disorders, including migraine and autoimmune disease, as well as genetic or familial influence (Frejo et al., 2016, 2017). More recent research has also identified autoinflammatory or allergic status as having an association with MD (Flook et al., 2023; Xu et al., 2023). Genetic analysis has revealed that mutations in *OTOG*, *MYO7A* and *TECTA*, as well as *CDH23*, *PCDH15* and *ADGRV1*, which are involved in stereocilia organisation and interaction with tectorial membrane, are associated with familial MD (Dai et al., 2023; Flook et al., 2023). The diverse and complex interactions between immune, genetic, fluid homeostasis and environmental factors likely contribute to this multifactorial disorder of the inner ear.

To date there is no cure for MD. An informative recent review describes in detail the various treatment options, ranging from conservative to destructive, that are currently recommended by five different international MD guidelines (Mohseni-Dargah et al., 2023). In all instances, at the end stage of disease, the last remaining treatment option requires either the removal of the peripheral vestibular organs or resection of the vestibular portion of the vestibulocochlear nerve. Excision of the vestibular organs in patients with MD is typically achieved by a translabyrinthine approach (Roberts et al., 2020). In surgical approaches involving the ablation of peripheral vestibular organs, drilling with irrigation and aspiration are characteristically used. However, for the tissue used in this study, the superficial aspect of each canal was drilled, and the membranous labyrinth and vestibular neuroepithelium were carefully removed via micro-hook or Fisch micro-raspatory. In previous studies, this tissue from patients with MD has predominantly been used for immunolabelling studies. However, owing to the proximity of our research laboratory and physiology expertise, we were able to use MD tissue samples for real-time electrophysiological experiments. To optimise the use of this valuable tissue, after electrophysiological recordings, samples were fixed and used for immunolabelling analysis. Here, we describe the feasibility of using surgically removed MD tissue for immunofluorescent labelling and electrophysiological recordings to investigate the expression of hair cell and afferent fibre proteins and to characterise voltage-gated currents in surviving vestibular hair cells in MD.

Many studies have used tissue samples following surgical removal, but some have also used cadaveric tissue from patients with MD (Stephenson et al., 2021; Calzada et al., 2012). Previous studies from surgically donated and cadaveric tissue have shown a loss of vestibular hair cells in patients with MD (Ishiyama et al., 2007; McCall et al., 2009). These reports using brightfield and transmission electron microscopy describe the loss of vestibular hair cell loss as highly variable between patients (Ishiyama et al., 2007; McCall et al., 2009). Other studies using post-mortem examinations of vestibular neuroepithelium in MD suggest that hair cell loss is highly variable, with preferential loss of type II vestibular hair cells and Scarpa's ganglion neurons (Tsuji et al., 2000). It is not known whether the surviving hair cells in diseased tissue are functional. We show in our study that surgically removed vestibular neuroepithelium can not only be used for anatomical investigations, but also functional studies. The majority of studies using adult human MD vestibular tissue have been histological, characterising and quantifying neuroepithelial components, including hair cells, supporting cells (Lopez et al., 2005), membranous labyrinth (Wackym et al., 1991) and basement membrane (McCall et al., 2009). Immunolabelling experiments describing proteins involved in endolymph recycling and extracellular matrix components (Lopez et al., 2019; Asmar et al., 2018; Calzada et al., 2012) have also been undertaken. More recently, studies have harvested inner ear tissue removed from patients with MD or vestibular schwannoma for investigations of regenerative capacity (Taylor et al., 2015) and for gene transfer studies (Kesser et al., 2007). These studies focus on the use of gene therapies to replace lost hair cells due to ageing, disease and damage (Taylor et al., 2015). There are very few studies that have recorded functional activity from human vestibular hair cells. Approximately 25 years ago, there were two studies that successfully took whole-cell patch-clamp recordings from human vestibular hair cells, although these were mostly from enzymatically dissociated cells (Oghalai et al., 1998, 2000). In addition, the recordings were voltage-activated currents from hair cells of patients with vestibular schwannoma. More recently, our laboratory has electrophysiologically recorded from human foetal vestibular hair cells in a semi-intact neuroepithelial preparation, which maintains the intracellular milieu (Lim et al., 2014). To date, there have not been any functional studies that have reported the viability of whole-cell patch-clamp recordings from vestibular hair cells from MD samples. As described above, there is variability in the survival of hair cells in MD. However, it is not known whether remaining vestibular hair cells are functional, or whether they are type I, type II or both.

Overall, there were two aims of this study: (1) to investigate the distribution of the hair cell-specific marker, myosin VIIa (MYO7A), and nerve fibre marker, neurofilament H, in the vestibular neuroepithelium from patients with MD and compare it to that in normal human vestibular neuroepithelial tissue; and (2) to confirm the viability of recording functional responses of surviving hair cells from MD neuroepithelium. Understanding whether surviving hair cells are functional is important to inform regenerative studies that target specific cell types for differentiation.

## RESULTS

In this preliminary study describing the feasibility of using surgically removed vestibular neuroepithelia for both electrophysiological and immunofluorescent studies, inner ear tissue was donated by three people with MD (age 56-68 years) and from two foetal tissue donors (12 and 14 weeks’ gestation). MD donors reported the length of time since diagnosis as between 3 and 10 years. Data from each figure represent an example from a single donor and might not represent all patients with MD, which have different clinical subtypes.

### Immunofluorescent labelling

**Fig. 1.**
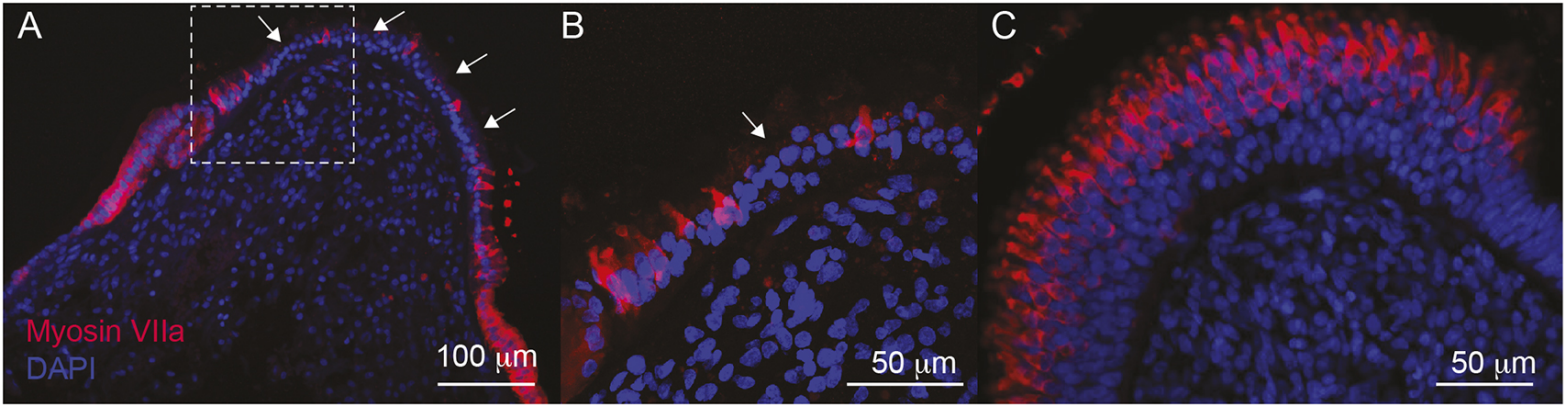
**Loss of vestibular hair cells in posterior crista from Ménière's disease (MD) tissue.** (A) Immunolabelling of the hair cell-specific marker, myosin VIIa (red), shows that few vestibular hair cells remain in the neuroepithelium in the MD sample. These are preferentially located at the periphery of the crista, with few in the central zone of the crista. There are large voids in which no hair cells are present (arrows). Cell nuclei are labelled with DAPI (blue). (B) High-magnification image of the region within the dashed outline in A. Few hair cells are present, and cell nuclei are disorganised within the neuroepithelium. A distinct supporting cell layer is absent. (C) Myosin VIIa immunoreactivity in the crista of a developing human foetal inner ear (12 weeks’ gestation) shows a high density of vestibular hair cells (red) throughout the neuroepithelium from the horizontal canal.

Here, we used immunofluorescence to label myosin VIIa in vestibular hair cells from a patient with MD. Fig. 1A,B shows the posterior crista from a patient with MD. Hair cells (Fig. 1A,B, red) are observed within the vestibular neuroepithelium. However, there are large voids, in which there are no hair cells present (Fig. 1A,B, arrows). This contrasts with normal developing human tissue (Fig. 1C), in which, even during early foetal development (12 weeks’ gestation), hair cells (Fig. 1C, red) are densely packed in the vestibular neuroepithelium.

**Fig. 2.**
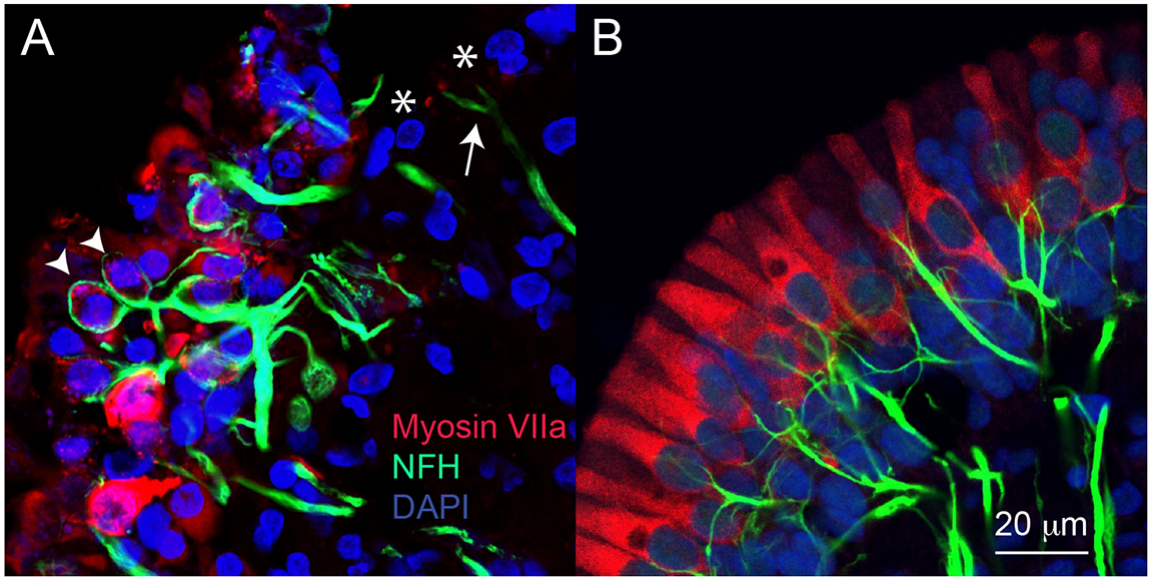
**Immunolabelling of hair cells and nerve fibres in MD neuroepithelium.** (A) Vestibular hair cells (myosin VIIa positive, red) are disorganised within the vestibular neuroepithelium of the anterior canal, with some areas devoid of hair cells (asterisks). Afferent nerve fibres [neurofilament H (NFH) positive, green] are present within the neuroepithelium and, in some instances, appear to surround or encapsulate hair cells (arrowheads). In regions of the neuroepithelium in which hair cells are no longer present (asterisks), afferent fibres appear thinner (arrow). Cell nuclei are labelled using DAPI (blue). (B) Hair cells (red) in the vestibular neuroepithelium from the anterior canal of the foetal inner ear (14 weeks’ gestation) show an organised sensory monolayer with some distinctions in morphology beginning to emerge. In human foetal tissue, afferent nerve fibres (green) penetrate the neuroepithelium and are beginning to contact hair cells. At this stage of development, no contacts resemble the characteristic calyceal amphora morphology surrounding type I vestibular hair cells. Developing afferent fibres in the developing human neuroepithelium are thinner than those observed in the adult MD neuroepithelium.

In Fig. 2A, immunolabelling of tissue from another sample with MD shows disorganised hair cells throughout the neuroepithelium of the anterior canal. There are regions in which hair cells are absent (Fig. 2A, asterisks) which contrasts with the dense packing of hair cells throughout the normal foetal neuroepithelium (Fig. 2B). MD tissue shows thick afferent nerve fibres penetrating the disorganised hair cell region, terminating as endings that surround or enclose assumed type I hair cells (Fig. 2A, arrowheads). In contrast, in regions in which hair cells are no longer present (Fig. 2A, asterisks), the afferent terminals appear thinner (Fig. 2A, arrow). In the developing human foetal neuroepithelium, the afferent fibres appear thinner than in the mature neuroepithelia of the MD sample. This is likely to be a function of development, and, at later stages of development, the fibres would increase in diameter. In the developing neuroepithelium, the afferent fibres are dispersed throughout the tissue and make contact with dense populations of hair cells (Fig. 2B). At this stage of foetal development (14 weeks’ gestation), there do not appear to be calyceal terminals that surround type I vestibular hair cells.

### Electrophysiological recordings from type I and II vestibular hair cells

**Fig. 3.**
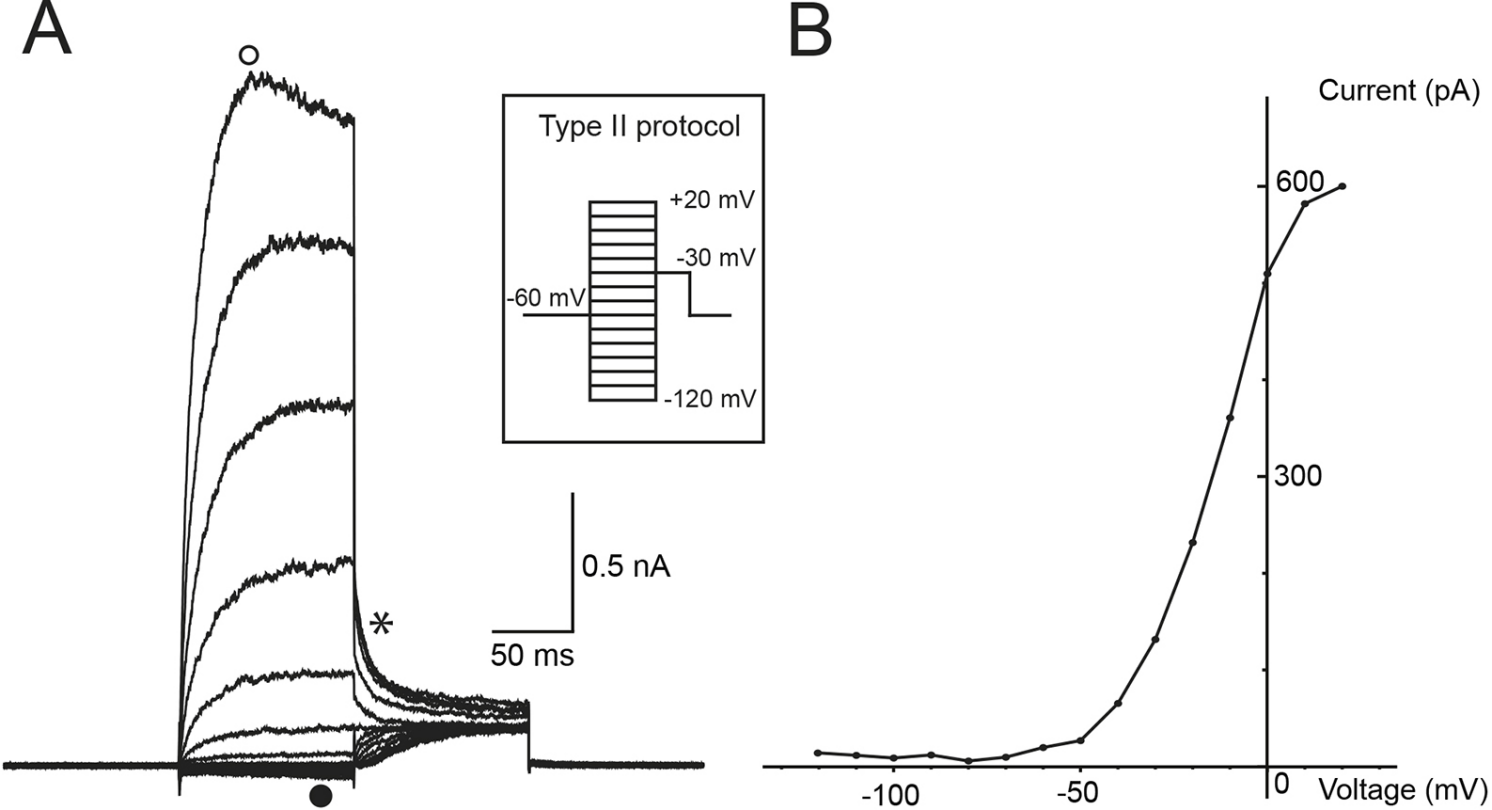
**Whole-cell patch-clamp recording of a type II vestibular hair cell from MD tissue.** (A) A voltage protocol (inset) is used to activate voltage-activated currents in a human type II vestibular hair cell from the utricle of a patient with MD. This type II vestibular hair cell exhibited very small inward currents at hyperpolarised potentials (‘•’) and large outward K^+^ currents at depolarised potentials (‘○’). Instantaneous tail currents (‘*’) were used to generate a I-V plot (B). (B) The activation plot from tail currents of a type II vestibular hair cell shown in A. Fitting the I-V curve using the Boltzmann equation calculates the maximum conductance (*G*_Max_) as 12.9 nS, *V*_½_ as −13.6 mV and slope as 11.5.

**Fig. 4.**
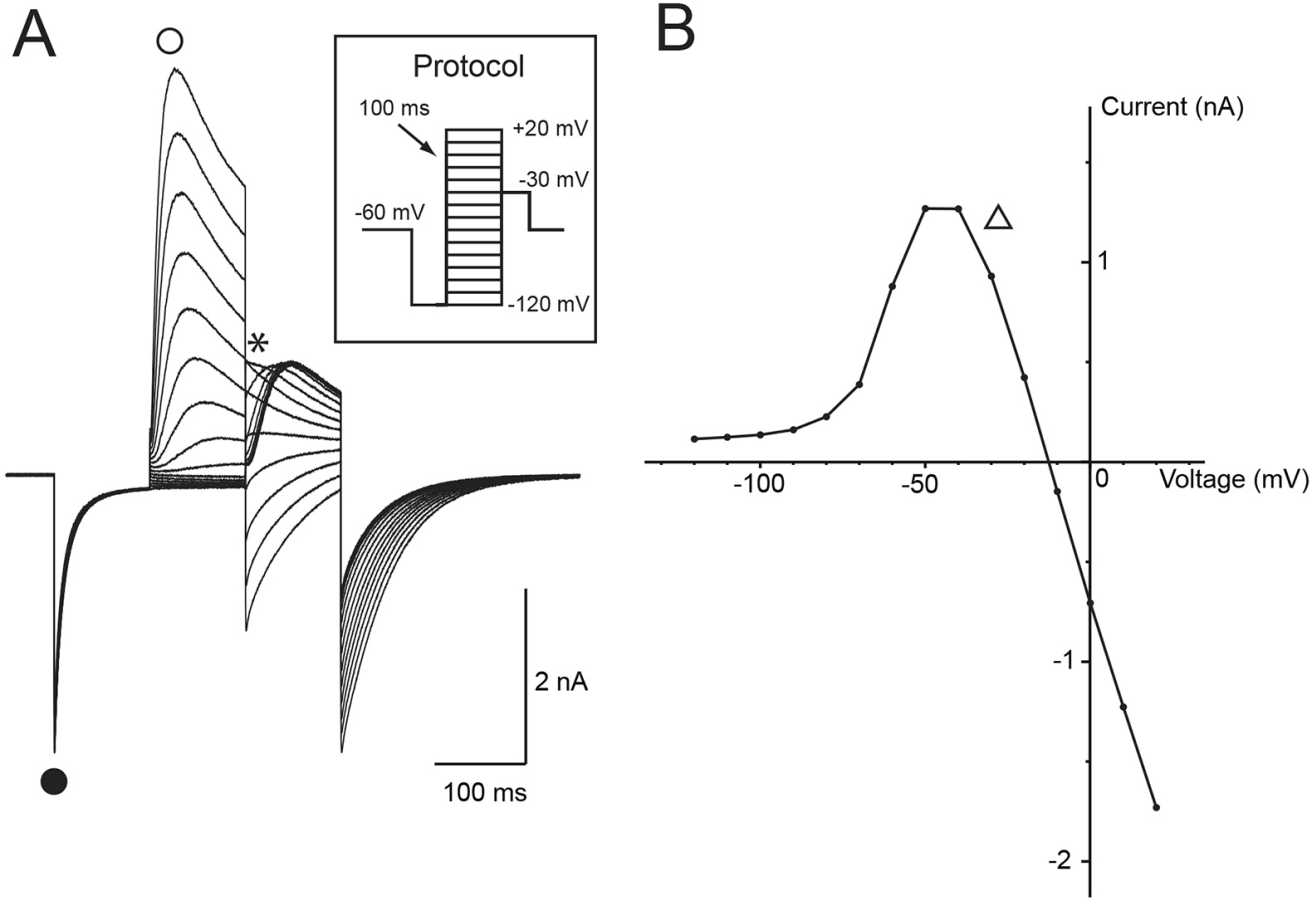
**Whole-cell patch-clamp recording of a type I vestibular hair cell from MD tissue.** (A) A voltage protocol (inset) is used to activate voltage-activated currents in a human type I vestibular hair cell. This protocol delivers a −120 mV hyperpolarising pre-pulse before the voltage ladder. This saccular type I vestibular hair cell had large inward currents at hyperpolarised potentials below −70 mV (‘•’), consistent with large low-voltage activated currents (*I*_K,L_) and very large outward currents at depolarised potentials (‘○’). Instantaneous tail currents (‘*’) were used to generate an activation I-V plot (B). (B) Activation I-V plot from tail currents of the type I vestibular hair cell shown in A. At voltages more positive than −40 mV, the currents begin to collapse (‘Δ’), and they reverse at ∼−15 mV.

The immunolabelling results above (Figs 1 and 2) show that there are significantly fewer vestibular hair cells in neuroepithelia from patients with MD than in normally developing human neuroepithelia. This made targeting vestibular hair cells for electrophysiological recordings challenging. Figs 3 and 4 show recordings from an utricular and saccular hair cell respectively. High-quality, stable electrophysiological recordings are required to calculate cellular properties such as maximum conductance (*G*_Max_), half-maximal activation potential (*V*_½_) and slope. It should be noted that taking electrophysiological recordings from tissue from MD donors presents significant technical challenges; for example, there are fewer hair cells to target within the neuroepithelium, aged peripheral nervous system tissue is notoriously difficult to record, and the surgical isolation procedure disrupts the natural fluid environment in which the tissue is bathed. These are all factors that significantly limit the number of viable electrophysiological recordings. Therefore, we are limited to reporting the values for these two macular organ hair cells only.

#### Type II vestibular hair cells

Whole-cell patch-clamp recordings were taken from MD neuroepithelium using the same technique as described in mouse (Lim et al., 2011) and developing human foetal vestibular neuroepithelium (Lim et al., 2014). Fig. 3A shows a recording from a type II utricular vestibular hair cell. These hair cells do not possess the large low-voltage-activated conductance (*G*_K,L_) that is characteristic of type I vestibular hair cells (Fig. 4, ‘•’). Type II vestibular hair cells exhibit variable inward rectifying currents (Fig. 3A, ‘•’) and large outward currents (Fig. 3A, ‘○’) in response to the voltage protocol shown in the inset in Fig. 3A. These currents are similar to those previously observed in mice (Lim et al., 2011) and humans (Lim et al., 2014). The activation I-V plot (Fig. 3B) for the cell's instantaneous tail currents (Fig. 3A, ‘*’) shows a characteristic sigmoidal I-V curve for a type II vestibular hair cell. Fitting the activation curve to this type II hair cells shows *G*_Max_ as 12.9 nS, *V*_½_ as −13.6 and slope as 11.5.

#### Type I vestibular hair cells

A type I vestibular hair cell recorded from the saccule in response to the voltage protocol (Fig. 4A, inset) shows large voltage-activated currents at hyperpolarised potentials (Fig. 4A, ‘•’), consistent with the presence of *G*_K,L_. During depolarising steps, the type I vestibular hair cell also exhibited large outward currents (Fig. 4A, ‘○’). These outward currents were significantly larger in amplitude than those recorded from the type II vestibular hair cell in Fig. 3A. An activation plot (Fig. 4B) taken from the instantaneous tail currents (Fig. 4A, ‘*’) of the type I hair cell shows the presence of characteristic collapsing activation as the hair cell is depolarised above −50 mV. This differs significantly from the classic sigmoidal activation curve observed in type II hair cells (Fig. 3B) and enzymatically isolated type I hair cells (Oghalai et al., 1998).

## DISCUSSION

In previous studies, we describe the procedures for microdissection of vestibular neuroepithelium and removal of overlying membrane for use in anatomical and functional experiments in mice and human foetal tissue (Lim et al., 2011, 2014). The same approach was used here. Our data show that, using a translabyrinthine approach, the surgical removal of vestibular organs from patients with MD provides tissue that can be used for both immunolabelling and electrophysiological investigation. Indeed, given the surprising robustness and viability of MD tissue for functional recordings, future work could also use cell culture techniques such as those previously described (Taylor et al., 2015) for additional longer-term investigations.

Consistent with previous anatomical data from deceased patients with MD (Ishiyama et al., 2015; McCall et al., 2009), our samples of MD vestibular neuroepithelia appear to exhibit the same severe hair cell loss and disorganised epithelia, which contrasts with the usual two distinct layers of hair cells and supporting cells. Previous research showed the merging into a single disorganised neuroepithelial layer primarily in the cristae ampullares, but less so in the utricular maculae (Ishiyama et al., 2015). Immunolabelling studies by others using MD tissue has shown distinct changes in the expression levels of proteins involved in inner ear fluid homeostasis, proton channels, basement membranes and extracellular matrix (Asmar et al., 2018; Calzada et al., 2012; Ishiyama et al., 2015; Lopez et al., 2019). Evidence suggests that proteins such as aquaporins, involved in inner ear fluid homeostasis, are modified in MD; reports have shown altered expression of aquaporins 4 and 6 in MD neuroepithelium compared to that in acoustic neuroma and post-mortem tissue from unaffected individuals (Ishiyama et al., 2015). Aquaporin 2 expression is elevated in the endolymphatic sac (Asmar et al., 2018), a site thought to be involved in the development of endolympatic hydrops, which is associated with MD. Expression of cochlin, a basement membrane component, is also significantly increased in MD (Calzada et al., 2012). Research from other laboratories (McCall et al., 2009; Okayasu et al., 2018), together with our results, shows that there is significant remodelling of the vestibular neuroepithelium and altered expression of various proteins involved in maintaining fluid homeostasis and neuroepithelial structural integrity in MD.

We show reduced labelling of afferent fibres by neurofilament H throughout the MD mesenchyme and neuroepithelium. This is consistent with results described in a suspected MD case, which also show reduced afferent fibres (Okayasu et al., 2018). However, cross-sectional ultrastructural analysis of MD nerve showed a very low percentage of abnormal nerve fibres (Kitamura et al., 1997). Similar non-pathological results examining the ultrastructure of vestibular nerve fibres observed at the level of the sensory epithelium and the internal acoustic meatus have been described (McCall et al., 2009). These ultrastructural studies, however, do not describe whether there is alteration in the number of afferent fibres in the neuroepithelium. Our preliminary findings describe reduced numbers of hair cells and afferent fibres in MD. Other studies have shown varying degrees of degeneration and lack of abnormal or pathological nerve fibres that remain in contact with surviving hair cells (McCall et al., 2009). The absence of sensory input is plausibly due to hair cell loss, which triggers the retraction and thinning of afferent fibres from the neuroepithelium. A similar process occurs in the auditory system after loss of inner hair cells, which results in degeneration of auditory nerve fibres from the sensory neuroepithelium (Shibata et al., 2011).

Even with considerable hair cell loss and disorganisation of the vestibular neuroepithelium, some hair cells remain. These surviving hair cells appear resistant to the overall changes induced by MD. We targeted these cells for electrophysiological characterisation. Our data show that hair cells from MD tissue display characteristic voltage-activated currents consistent with those of type I and type II vestibular hair cells. *G*_Max_, *V*_1/2_ and slope values for the type II vestibular hair cell are consistent with those previously recorded from human tissue, albeit foetal in origin (Lim et al., 2014). One other group has electrophysiologically recorded from adult human vestibular hair cells (Oghalai et al., 1998), although these were isolated vestibular hair cells, and none were from patients with MD. We developed a semi-intact vestibular explant in mouse (Lim et al., 2011) and human (Lim et al., 2014), which maintains a more normal intracellular milieu, for electrophysiological recording of hair cells. Maintenance of this intracellular milieu reveals a complex interaction of hair cells and their calyx afferent terminals (Lim et al., 2011; Contini et al., 2020). Indeed, maintenance of this intracellular milieu between type I hair cells and their associated calyx afferent terminal results in activation curves that ‘collapse’ (Lim et al., 2011; Contini et al., 2012). Potassium ions released from basolateral channels of the type I hair cells accumulate in the surrounding gap between hair cell and calyx terminal and significantly alter recording conditions. Thus, the presence of a collapsing activation curve in Fig. 4 is indicative of a calyx afferent terminal surrounding a type I hair cell in the MD sample. These collapsing tail currents are only observed in semi-intact neuroepithelial preparations in which the intracellular milieu is maintained, and not in dissociated hair cell recordings.

Our recordings from MD tissue provide evidence of both type II and type I vestibular hair cells surrounded by a calyx afferent terminal. This suggests that MD does not preferentially result in the death of a particular hair cell type. Gaining an understanding of whether the hair cell–afferent fibre connection remains functional in MD is important for establishing which neural elements need to be targeted in regenerative and neuro-prosthetic studies. Recent regeneration studies have shown that supporting cells in the vestibular neuroepithelium have the capacity for trans-differentiation into functional type II vestibular hair cells that express type II hair cell-specific whole-cell currents and are capable of transducing bundle deflections (Gonzalez-Garrido et al., 2021; Hicks et al., 2020). However, there was no evidence of regeneration of type I vestibular hair cells (Gonzalez-Garrido et al., 2021; Hicks et al., 2020). Further work is required to discover the signalling pathways that underlie the differentiation of vestibular type I hair cells that are essential for restoration of vestibular function. Most importantly, however, we still need to determine the underlying cause of MD. Without addressing the cause of MD, treatment for this disorder, including regeneration of vestibular hair cells, is likely to be compromised, and patients will continue to experience severe vertigo and dizziness.

Further work is also required to establish the capacity of remaining vestibular afferent fibres to discharge action potentials in the presence of MD. If vestibular afferent fibres remain viable, there is enormous potential to stimulate these fibres using a vestibular implant in the same manner as cochlear implants stimulate cochlear afferent fibres to restore hearing. It needs to be acknowledged that vestibular implants are likely to be most effective at the late stage or ‘burn-out’ of MD, when hair cells are absent but afferent fibres remain to transmit signals to the central nervous system. However, the loss of vestibular sensory input, like that encountered during peripheral auditory deafness, could result in degeneration of afferent fibres, which in turn would limit the effectiveness of vestibular implants. This suggests that an optimal temporal window for implantation would need to be determined for the implant signal to be transmitted via functional and viable vestibular afferent fibres. Considerable work still needs to be done to understand the cellular changes that occur in MD and for the development of appropriate restoration and implant technologies. The ability to restore balance function in people with MD would result in significant improvement in their quality of life.

## MATERIALS AND METHODS

### Ethical considerations

All research in this study was approved by Hunter New England Health Ethics Committee or the University of Newcastle Human Research Ethics Committee, and all experimental work was approved by University of Newcastle Institutional Biosafety Committee, followed the Standards for Reporting Qualitative Research (SRQR) guidelines (O'Brien et al., 2014) and was performed according to the ethical guidelines set forth in the Declaration of Helsinki. We report on the feasibility of using diseased human vestibular tissue for electrophysiological recordings. Therefore, the results shown here are representative of the quality of data that can be acquired from tissue from people affected by MD. Written consent was obtained from donors prior to surgery. The surgical and research teams worked independently. The surgical team was not involved in collection of electrophysiological data, and research team members were not involved in surgical procedures. Data shown are from MD tissue that required surgical removal of vestibular organs for symptom treatment. Surgeries were performed in local Newcastle hospitals in New South Wales, Australia. Specimens were collected from surgical procedures performed by one clinician. Consequently, limited data have been reported to ensure donor privacy.

All patient data were de-identified and stored securely. Two-factor authentication was required by researchers to access patient data, which included sex, date of birth and length of time since diagnosis. No other personal patient data were collected.

### Tissue collection and transport

The translabyrinthine approach was used to remove vestibular organs (Roberts et al., 2020). Briefly, the superficial aspect of each semicircular canal was drilled to expose the membranous labyrinth. A Fisch micro-raspatory (Aesculap Surgical Instruments, Braun, Germany) was used to retrieve the vestibular neuroepithelium from the ampullae of the semicircular canals and the utricle and saccule from the vestibule. The neuroepithelium was then collected in cold Liebovitz's cell culture medium (Life Technologies, Australia), which contained the following; 1.26 mM CaCl_2_, 0.99 mM MgCl_2_, 0.81 mM MgSO_4_, 5.33 mM KCl, 0.44 mM KH_2_PO_4_, 137.93 mM NaCl, 1.34 mM Na_2_PO_4_, pH 7.45, 305 mOsM). A research team member ransported the tissue samples to the laboratory at The University of Newcastle. The samples were inspected for viability and used for electrophysiological and/or immunolabelling experiments, depending on the tissue quality. All recovered vestibular neuroepithelial tissue was inspected under a dissecting microscope to determine whether it could be used initially for electrophysiological recording followed by immunofluorescent labelling studies. If the tissue was physically damaged, torn or contained blood, it was not used for electrophysiological studies but fixed with paraformaldehyde. The fixed tissue was then further evaluated to determine whether it could be used for immunolabelling studies (see ‘Tissue preparation: immunofluorescent labelling’ section). Foetal samples were collected and prepared as described previously (Lim et al., 2014).

### Tissue preparation: immunofluorescent labelling

Samples for immunofluorescent labelling were fixed in 4% paraformaldehyde (0.1 M PBS) overnight and then washed in 0.1 M PBS and stored in 0.1 M PBS and 0.05% sodium azide until processing. Neuroepithelial tissue was cryoprotected in 30% sucrose (0.1 M PBS) overnight, and sections (20-50 µm) were cut using a CM1950 cryostat (Leica Microsystems, VIC, Australia). Primary antibodies against markers of hair cells (rabbit anti-myosin VIIa, 1:200, Proteus Biosciences, MA, USA, 25-6790) and afferent fibres (anti-neurofilament H, 1:500, Merck Life Sciences, VIC, Australia, AB5539) were used for immunofluorescent labelling. Sections were incubated overnight in primary antibodies then washed (3×0.1 M PBS) and incubated in secondary antibodies conjugated to Alexa Fluor 594 (1:200) or Alexa Fluor 488 (1:200) (both from Abcam, Cambridge, UK, ab150112 and ab150173, respectively) for 2 h. DAPI (1:1000, Merck Life Sciences) was used to label cell nuclei. Sections were mounted using 0.1 M PBS/50% glycerol and cover-slipped. Images were captured using either a Nikon Eclipse EZ1 confocal microscope or Zeiss Axio Observer confocal microscope.

### Tissue preparation: electrophysiology

Tissue was prepared as previously described (Lim et al., 2014). In brief, bone debris and membranes overlying the vestibular neuroepithelia were removed using fine forceps and curved micro-scissors. This procedure was done in cold (4°C) Liebovitz's L15 cell culture medium (Life Technologies, Australia). The individual vestibular neuroepithelia were further isolated, secured in place by a stringed harp in a recording chamber containing fresh, room-temperature, L-15 solution. The recording chamber (volume, 1 ml; flow rate, 2 ml/min) was perfused with oxygenated Liebovitz's cell culture medium.

### Electrophysiological recordings

Borosilicate glass microelectrodes (3-5 MOhm; King Precision Glass, CA, USA) were pulled using a Narishige electrode puller (PC-10) and filled with potassium gluconate internal solution [containing 42 mM KCl and 1 mM MgCl_2_ (both Thermo Fisher Scientific, MA, USA), 98 mM potassium gluconate, 4 mM HEPES, 0.5 mM EGTA and 5 mM sodium ATP (all from Merck Life Sciences, VIC, Australia), pH 7.3, 290 mOsM]. Electrophysiological recordings were made by targeting cells under a Zeiss Axioskop 2 microscope using infra-red differential interference contrast optics. Recordings were made using an Axopatch 200B amplifier, and data were acquired using Axograph X software (sampled at 20 kHz and filtered at 2-10 kHz). Whole-cell voltage-clamp protocols were used to characterise cell type. To determine cell type, a type I hair cell protocol was first used to establish whether the cell possesses the type I hair cell-specific *G*_K,L_ conductance (Fig. 3, inset). If a cell did not have *G*_K,L_ conductance, a type II hair cell protocol was used (Fig. 4, inset). This is identical to the type I hair cell protocol, with the exception that there is no hyperpolarising step to −120 mV (Fig. 3, inset).

After electrophysiological recordings were complete, the samples were fixed in 4% paraformaldehyde overnight, then washed and stored in 0.1 M PBS/sodium azide. These samples were also used for immunolabelling studies.
